# IL-13 induces loss of CFTR in ionocytes and reduces airway epithelial fluid absorption

**DOI:** 10.1172/JCI181995

**Published:** 2024-09-10

**Authors:** Guillermo S. Romano Ibarra, Lei Lei, Wenjie Yu, Andrew L. Thurman, Nicholas D. Gansemer, David K. Meyerholz, Alejandro A. Pezzulo, Paul B. McCray, Ian M. Thornell, David A. Stoltz

**Affiliations:** 1 Department of Internal Medicine,; 2 Department of Pediatrics,; 3 Department of Pathology,; 4 Pappajohn Biomedical Institute, and; 5 Department of Molecular Physiology and Biophysics, Roy J. and Lucille A. Carver College of Medicine, University of Iowa, Iowa City, Iowa, USA.; 6 Department of Biomedical Engineering, University of Iowa, Iowa City, Iowa, USA.

**Keywords:** Pulmonology, Asthma, Chloride channels, Th2 response

## Abstract

The airway surface liquid (ASL) plays a crucial role in lung defense mechanisms, and its composition and volume are regulated by the airway epithelium. The cystic fibrosis transmembrane conductance regulator (CFTR) is abundantly expressed in a rare airway epithelial cell type called an ionocyte. Recently, we demonstrated that ionocytes can increase liquid absorption through apical CFTR and basolateral barttin/chloride channels, while airway secretory cells mediate liquid secretion through apical CFTR channels and basolateral NKCC1 transporters. Th2-driven (IL-4/IL-13) airway diseases, such as asthma, cause goblet cell metaplasia, accompanied by increased mucus production and airway secretions. In this study, we investigate the effect of IL-13 on chloride and liquid transport performed by ionocytes. IL-13 treatment of human airway epithelia was associated with reduced epithelial liquid absorption rates and increased ASL volume. Additionally, IL-13 treatment reduced the abundance of CFTR-positive ionocytes and increased the abundance of CFTR-positive secretory cells. Increasing ionocyte abundance attenuated liquid secretion caused by IL-13. Finally, CFTR-positive ionocytes were less common in asthma and chronic obstructive pulmonary disease and were associated with airflow obstruction. Our findings suggest that loss of CFTR in ionocytes contributes to the liquid secretion observed in IL-13–mediated airway diseases.

## Abstract 2

Our findings suggest that loss of CFTR in ionocytes in Th2 airway diseases is associated with disease pathogenesis.

## Introduction

The airway surface liquid (ASL) that overlies the respiratory tract epithelium is an important component of host defense. The composition of this thin layer of liquid is tightly regulated and maintains ciliary function, mucus biophysical properties, and mucociliary transport (1–4). ASL also contains antimicrobial proteins and peptides that are important in the lung’s innate defense system (5, 6). Changes in ASL pH and volume have been implicated in airway diseases including cystic fibrosis (CF), asthma, and chronic obstructive pulmonary disease (COPD) (2, 5, 7–10).

Interleukin-13 (IL-13) is an inflammatory cytokine that plays a role in allergic inflammation often seen in Th2 inflammatory responses (11–14). Individuals with asthma and COPD are more likely to have elevated levels of IL-13 (11, 12), and these diseases are typically characterized by mucus hypersecretion and goblet cell hyperplasia (15–18). Previous studies using human tracheobronchial epithelial cells cultured at the air-liquid interface have shown that Th2 cytokines, IL-4 and IL-13, induce goblet cell hyperplasia and mucus hypersecretion (14, 19–26). Further, inhibition of IL-13–mediated signaling can reverse goblet cell hyperplasia (27, 28), suggesting that IL-13 may contribute to the pathophysiology of airway diseases such as asthma and COPD.

Despite accounting for approximately 1% of the cells in the airway, ionocytes express very high levels of cystic fibrosis transmembrane conductance regulator (CFTR) channels compared with secretory cells (29–32). We recently reported that whereas CFTR channels in human airway epithelial secretory cells increase transepithelial liquid secretion, CFTR channels in ionocytes increase liquid absorption across healthy airways (33), suggesting that cellular segregation of CFTR channels allows for control of ASL volume. Th2 inflammatory responses associated with subsets of asthma and COPD increase both Cl^–^ and mucus secretion (9, 13, 34, 35). How Th2 inflammatory responses, which increase Cl^–^ and mucus secretion through secretory cells, affect ionocytes is unknown. In this study, we investigated how IL-13 impacts airway liquid handling and whether IL-13 treatment affects ionocyte number and/or function.

## Results

### IL-13 exposure increases goblet cell abundance and Cl^–^ secretion.

**Figure 1:**
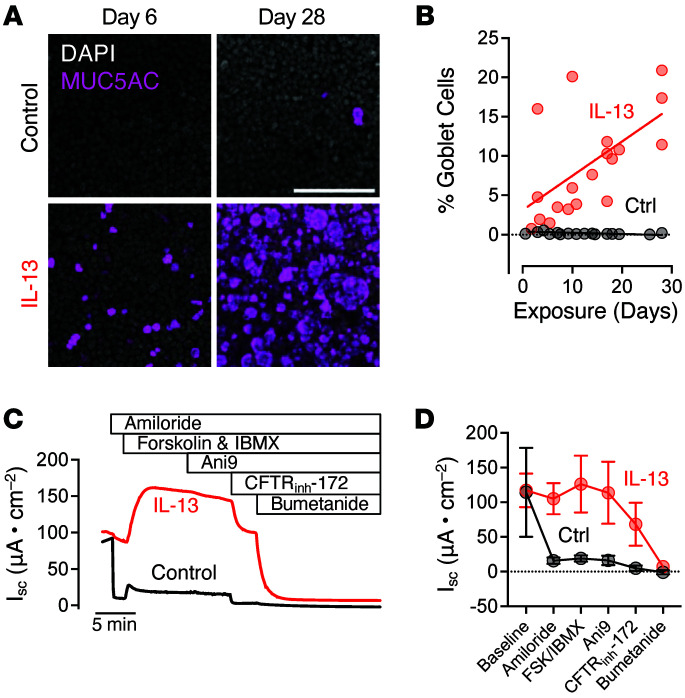
IL-13 treatment increases airway epithelial goblet cell number and bumetanide-sensitive *I*_sc_. (**A**) MUC5AC immunostaining of human airway epithelial cells treated with vehicle control or IL-13 for 6 or 28 days. Scale bar: 100 μm. (**B**) Quantification of goblet cell abundance (MUC5AC-positive cells) by flow cytometry at various time points following vehicle control or IL-13 treatment. Data are from 8 independent donors. Each symbol represents an individual donor at a given time point. Not all donors are represented at each time point. (**C**) Representative short-circuit current (*I*_sc_) traces of human airway epithelial cultures exposed to vehicle control or IL-13. The following agents were added sequentially: apical amiloride, apical forskolin/IBMX, apical Ani9, apical CFTR_inh_-172, and basolateral bumetanide. *n* = 5 donor epithelia per treatment group. (**D**) Summary *I*_sc_ values for donor epithelia treated with vehicle control or IL-13 for 3–4 weeks. Ctrl, control.

Treating airway epithelia with IL-13 increased the number of MUC5AC-positive goblet cells (Figure 1, A and B). We evaluated the effect of IL-13 on CFTR-mediated Cl^–^ secretion using the short-circuit current (*I*_sc_) technique (Figure 1C). Adding the epithelial Na^+^ channel (ENaC) inhibitor amiloride induced a larger fall in *I*_sc_ for control epithelia but only a minor decrease for IL-13 conditions (Figure 1, C and D). The change in *I*_sc_ (Δ*I*_sc_) with forskolin and IBMX was greater for IL-13–treated epithelia compared with controls (Figure 1, C and D). Similarly, TMEM16A inhibition, with Ani9, and CFTR inhibition, with CFTR_inh_-172, caused a greater Δ*I*_sc_ for IL-13–treated compared with untreated epithelia (Figure 1, C and D). Inhibition of the basolateral NKCC1 transporter with bumetanide dissipated the remaining *I*_sc_ (Figure 1, C and D). Therefore, IL-13 treatment increased transepithelial Cl^–^ secretion. The large residual *I*_sc_ after amiloride treatment produced by IL-13–treated epithelia (Figure 1, C and D) indicated that IL-13 altered the driving force for Na^+^ across ENaC.

The post-amiloride increase in *I*_sc_ with IL-13 depended on CFTR, since IL-13 failed to increase post-amiloride *I*_sc_ values when CF epithelia were studied (Supplemental Figure 1, A and B; supplemental material available online with this article; https://doi.org/10.1172/JCI181995DS1). Furthermore, in these experiments and additional experiments performed on non-CF epithelia bathed in nominally Cl^–^-free solutions, IL-13 did not affect amiloride-sensitive *I*_sc_ values, suggesting that the large increase in Cl^–^ channel function produced the difference in amiloride-sensitive *I*_sc_ observed after treatment of non-CF epithelia with IL-13 (Supplemental Figure 1, A–D).

### IL-13 treatment reduces barttin-positive ionocyte abundance.

**Figure 2:**
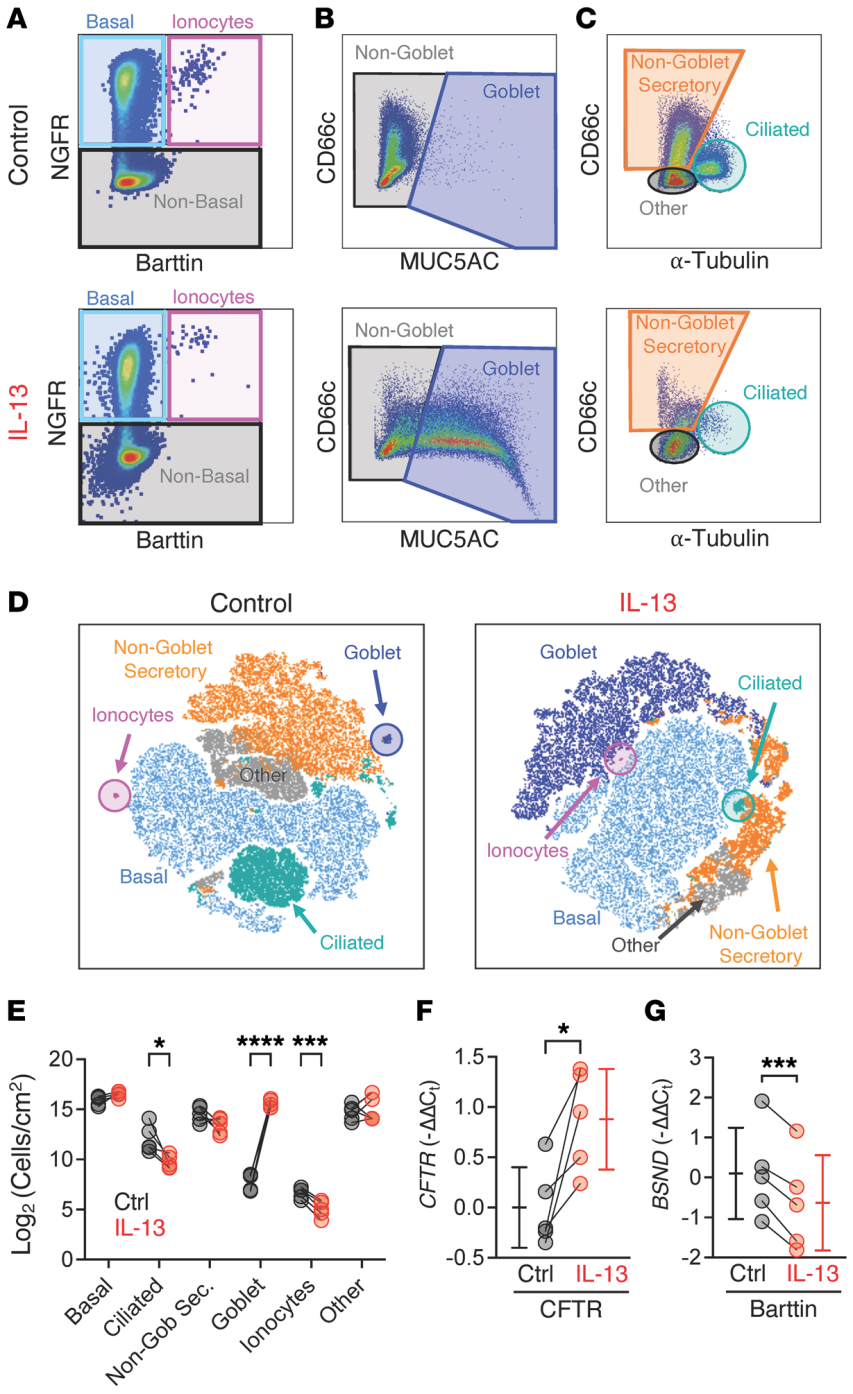
IL-13 reduces barttin-positive ionocyte abundance in airway epithelia. Human airway epithelial cultures were dissociated and analyzed by multicolor flow cytometry. (**A**–**C**) Top row represents vehicle control epithelia, and lower row represents epithelia treated with IL-13 for 3 weeks. (**A**) Panels are gated on live singlets and show NGFR detection levels by barttin (BSND) levels to identify basal cells (teal) and ionocytes (pink). (**B**) Panels are gated on gray gate in **A** (NGFR-negative, barttin-negative) and depict CD66c detection by MUC5AC detection. Goblet cells are indicated by dark blue gate. (**C**) Panels are gated on non-goblet cells (MUC5AC-negative) gray gate in **B** and show cells by detection of CD66c versus α-tubulin. Ciliated cells are α-tubulin–positive. (**D**) User-defined gates from flow cytometric data graphed in **A**–**C** displayed as a t-distributed stochastic neighbor embedding (t-SNE) plot. (**E**) Cell type abundance quantified from flow cytometry data in vehicle control (gray symbols) or IL-13–treated (red symbols) epithelia. (**F** and **G**) Total epithelial transcript levels for *CFTR* (**F**) and *BSND* (gene encoding barttin) (**G**). Data points connected by a line represent paired experiments from a single human donor. Data are shown as mean ± SD. *P* values were obtained using a 2-way ANOVA with multiple comparisons or paired, 2-sided Student’s *t* test. Ctrl, control. *P < 0.05; ***P < 0.001; ****P < 0.0001.

In healthy human airway epithelia, Lei et al. found that CFTR channels on secretory cells participate in Cl^–^ secretion while ionocytes absorb Cl^–^ through apical CFTR channels and ionocyte-specific basolateral barttin/Cl^–^ channels (33). To evaluate how these barttin-containing ionocytes respond to IL-13, we optimized a multicolor flow cytometry approach to determine the cellular composition of airway epithelia (Figure 2, A–C, and Supplemental Figure 2). IL-13 treatment increased the number of goblet cells and reduced ciliated cell abundance (Figure 2, D and E). Surprisingly, we detected about half as many barttin-positive ionocytes in IL-13–treated epithelia compared with controls (Figure 2E). At the transcript level, IL-13 increased epithelial *CFTR* expression, yet decreased epithelial *BSND*, which encodes ionocyte-specific barttin (Figure 2, F and G). These data suggest that IL-13 increases CFTR in secretory cells while simultaneously decreasing the absorption function of ionocytes.

### IL-13 increases the number of CFTR-positive goblet cells, but decreases the number of CFTR-positive ionocytes.

**Figure 3:**
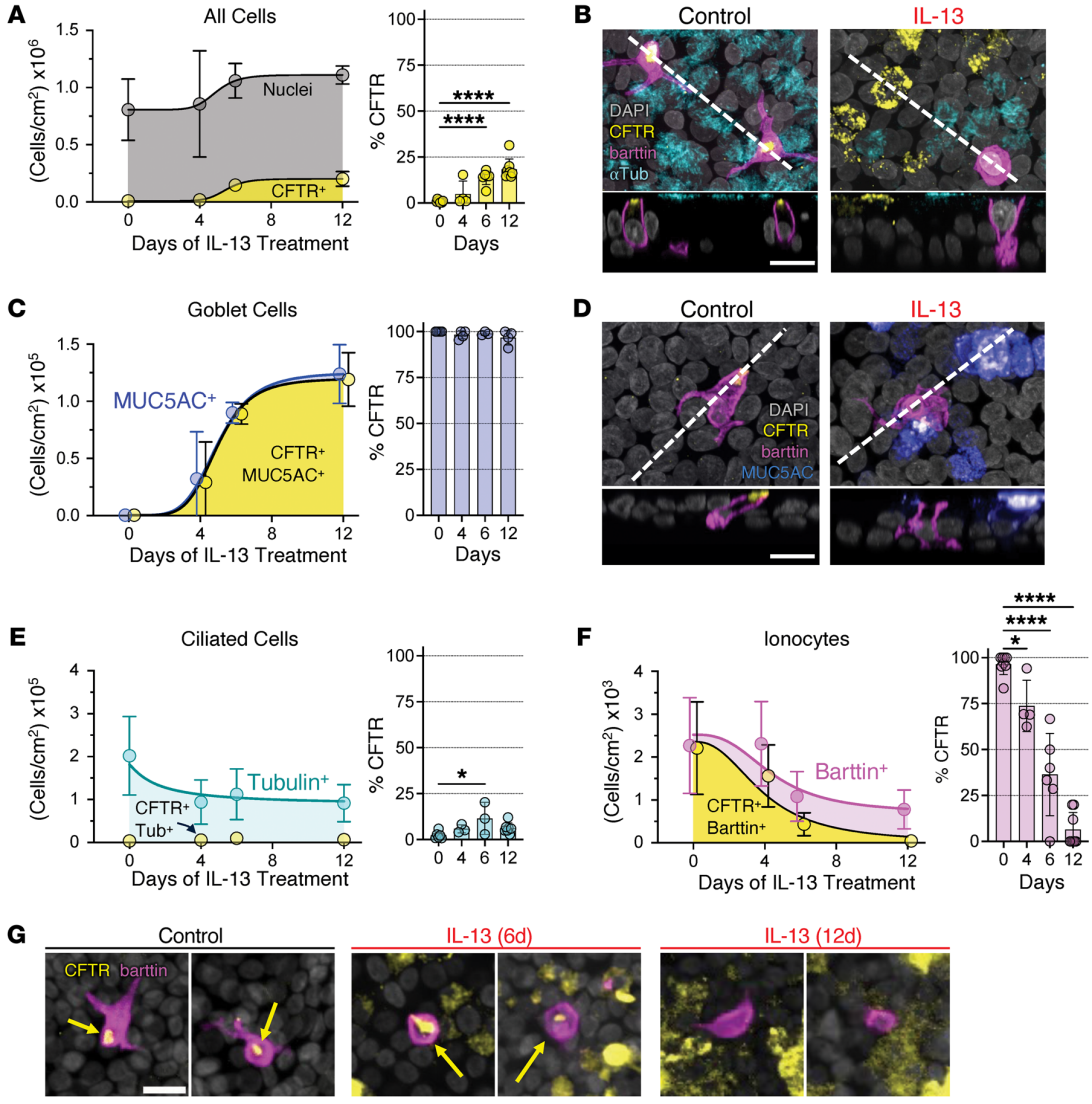
IL-13 rapidly reduces CFTR detection on ionocytes and increases goblet cell abundance. Immunofluorescence quantification and representative confocal microscopy images of different cell types and percentage of CFTR-positive cells over time in vehicle control and IL-13–treated epithelia. (**A**) Quantification of all cell types (DAPI-positive) and CFTR detection. (**B**) Epithelia were defined by DAPI (gray, all nuclei), CFTR (yellow), barttin (magenta, ionocytes), and α-tubulin (cyan, ciliated cells). (**C**) Quantification of goblet cells (MUC5AC-positive) and CFTR detection. (**D**) Epithelia were defined by DAPI (gray, all nuclei), CFTR (yellow), barttin (magenta, ionocytes), and MUC5AC (cyan, goblet cells). (**E**) Quantification of ciliated cells (α-tubulin–positive) and CFTR detection. (**F**) Quantification of ionocytes (barttin-positive) and CFTR detection. (**G**) Epithelia were defined for CFTR (yellow) and barttin (magenta, to identify ionocytes). Yellow arrows denote CFTR-positive ionocytes. Data are shown as mean ± SD. For percentage CFTR graphs, each symbol represents an independent donor. *P* values were obtained using an ordinary 1-way ANOVA with Dunnett’s multiple-comparison test between treatment time points versus day 0 controls. *n* = 3–7 independent human donors. (**B** and **D**, top panels, and **G**): *XY* projection. (**B** and **D**, bottom panels): *Z* projections taken at the dashed line and shown below the *XY* image. Scale bars: 15 μm. *P < 0.05; ****P < 0.0001.

Finding an increase in *CFTR* transcripts and a decrease in *BSND* transcripts and barttin-positive ionocytes suggested that secretory cells drove the increase in CFTR. To test this hypothesis, we performed immunofluorescence confocal microscopy after a time course of IL-13 exposures to investigate which cell types express CFTR. IL-13 exposure increased the total number of cells and cells with CFTR within 6 days (Figure 3, A and B). CFTR was nearly always observed on goblet cells, and IL-13 increased the number of goblet cells as early as day 4 (Figure 3, C and D). Accompanying the increase in goblet cells was a decrease in ciliated cells (Figure 3E). We rarely detected CFTR in ciliated cells (Figure 3, B and E). IL-13 treatment decreased the number of barttin-positive ionocytes and CFTR detection in remaining ionocytes as early as 4 days, and CFTR was rarely detected in ionocytes by day 12 (Figure 3, B, F, and G). Using fluorescence in situ hybridization, we found that IL-13 treatment reduced *CFTR* mRNA expression on ionocytes (Supplemental Figure 3). These results and our flow cytometry data indicate that an increase in CFTR-expressing goblet cells drives the increase in CFTR during IL-13 exposure, and any remaining barttin-positive ionocytes lack CFTR.

### IL-13 treatment reduces transepithelial liquid absorption.

**Figure 4:**
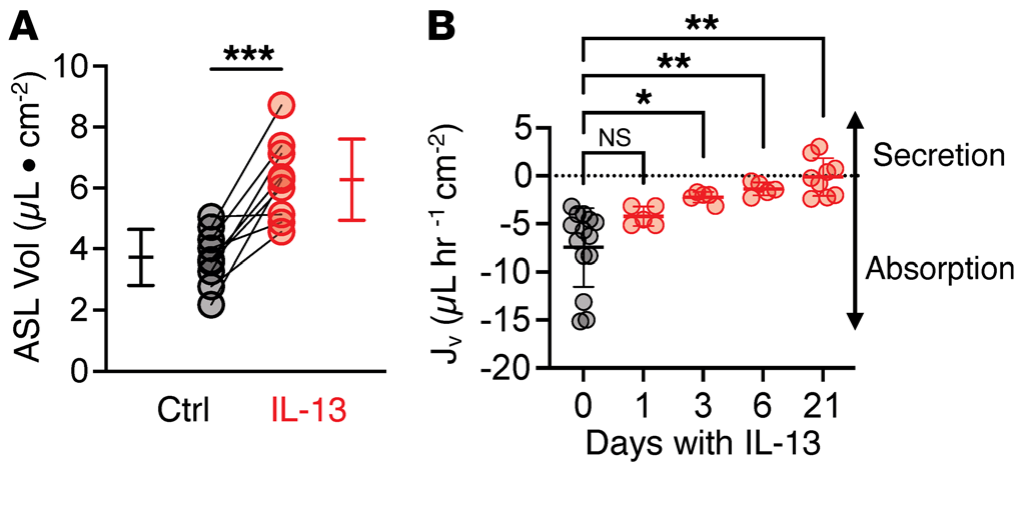
IL-13 treatment decreases airway epithelial liquid absorption. (**A**) ASL volume of control and IL-13–treated human airway epithelial cultures. (**B**) Liquid flux (*J*_v_) in epithelial cultures at various time points after IL-13 exposure and their respective controls. *n* = 5–14 independent human donors. Data are shown as mean ± SD. *P* values were obtained by a paired 2-tailed *t* test or mixed-effects analysis using Dunnett’s multiple-comparison test comparing treatment time points with controls (day 0). Ctrl, control. *P < 0.05; **P < 0.01; ***P < 0.001.

Since IL-13 decreased CFTR and barttin in ionocytes, we hypothesized that IL-13 would decrease liquid absorption. We tested this hypothesis using 2 approaches. First, we measured ASL volume and found that IL-13 treatment significantly increased ASL volume in cultured epithelium, a finding consistent with reduced transepithelial liquid absorption (Figure 4A). Second, we directly assayed the epithelial liquid absorption rate by adding saline to the apical epithelial surface and 4 hours later measuring the recovered liquid on the apical surface (33, 36–38). Under control conditions, human airway epithelia are absorptive (36–38). Therefore, we predicted that IL-13 treatment would reduce liquid absorption. IL-13–treated epithelia had a decreased liquid absorption rate, and we occasionally observed liquid secretion (Figure 4B). This shift toward a liquid-secretion phenotype, as indicated by a decrease in absorption, began as early as 3 days (Figure 4B). Thus, IL-13 created a less absorptive epithelium, consistent with our observation that after IL-13 exposure most epithelial CFTR channels exist in secretory cells where CFTR mediates Cl^–^ secretion.

### Increasing ionocyte abundance limits IL-13–induced liquid secretion.

**Figure 5:**
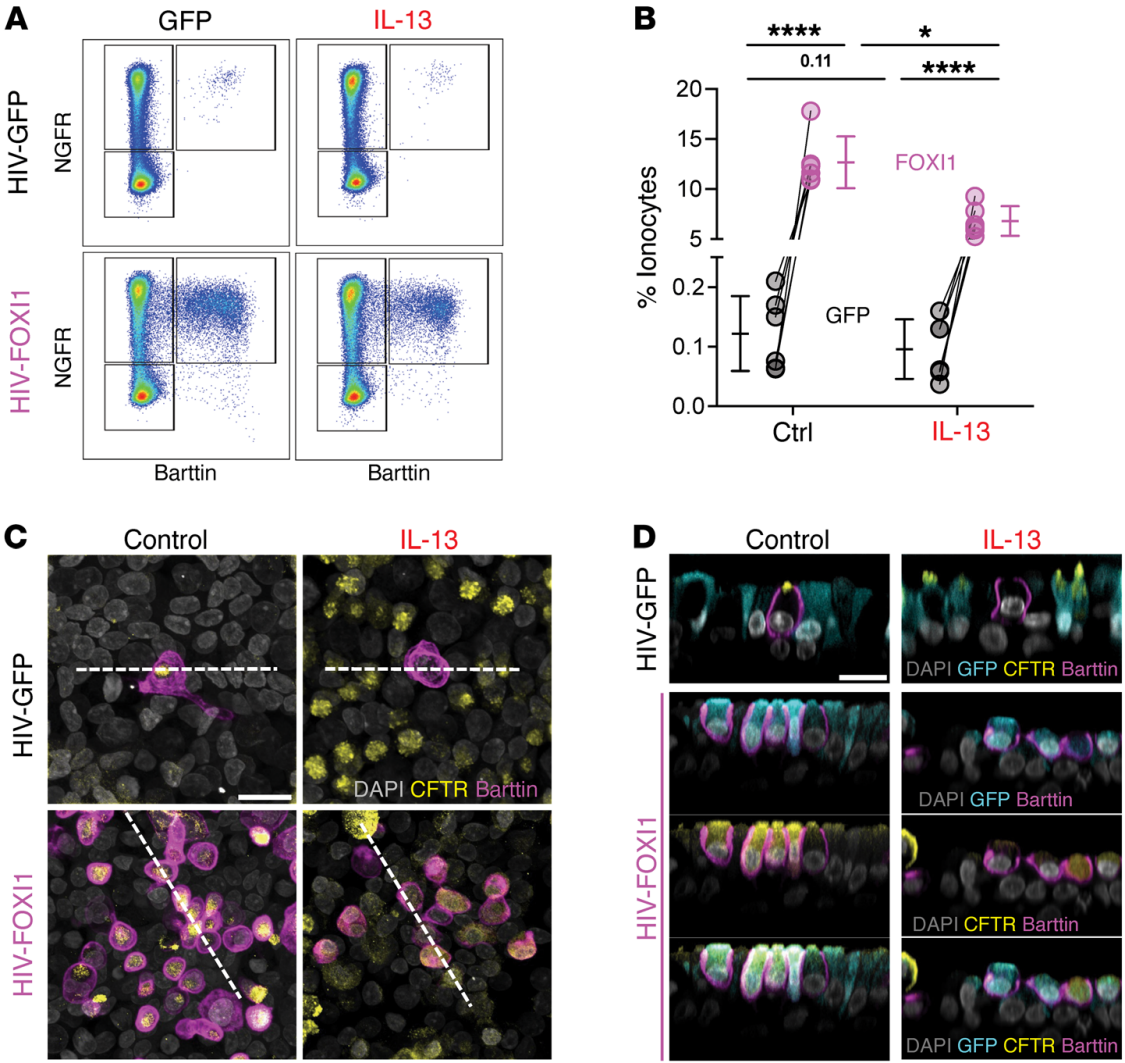
Overexpressing FOXI1 increases ionocyte abundance in IL-13–treated epithelia. Human airway epithelia were transduced with a lentivirus expressing GFP or FOXI1 and GFP, to generate and label ionocytes, respectively. Epithelia were then treated with vehicle control or IL-13 for 24 days. (**A**) Representative flow cytometry plots showing live, single cells. Cells are plotted by NGFR and barttin detection. (**B**) The relative abundance (percent) of ionocytes (NGFR-positive, barttin-positive events) within live single cells using flow cytometry. Data points connected by a line represent paired experiments from a single human donor. Data are shown as mean ± SD. (**C**) Confocal images of airway epithelia stained with DAPI (gray, all nuclei), CFTR (yellow), and barttin (magenta, ionocytes). Scale bar: 15 μm. (**D**) *XZ* projections of the confocal images in **C** taken at the dashed lines showing DAPI (gray, all nuclei), CFTR (yellow), barttin (magenta, ionocytes), and GFP (green). Scale bar: 15 μm. Ctrl, control; GFP, green fluorescent protein. *P < 0.05; ****P < 0.0001.

**Figure 6:**
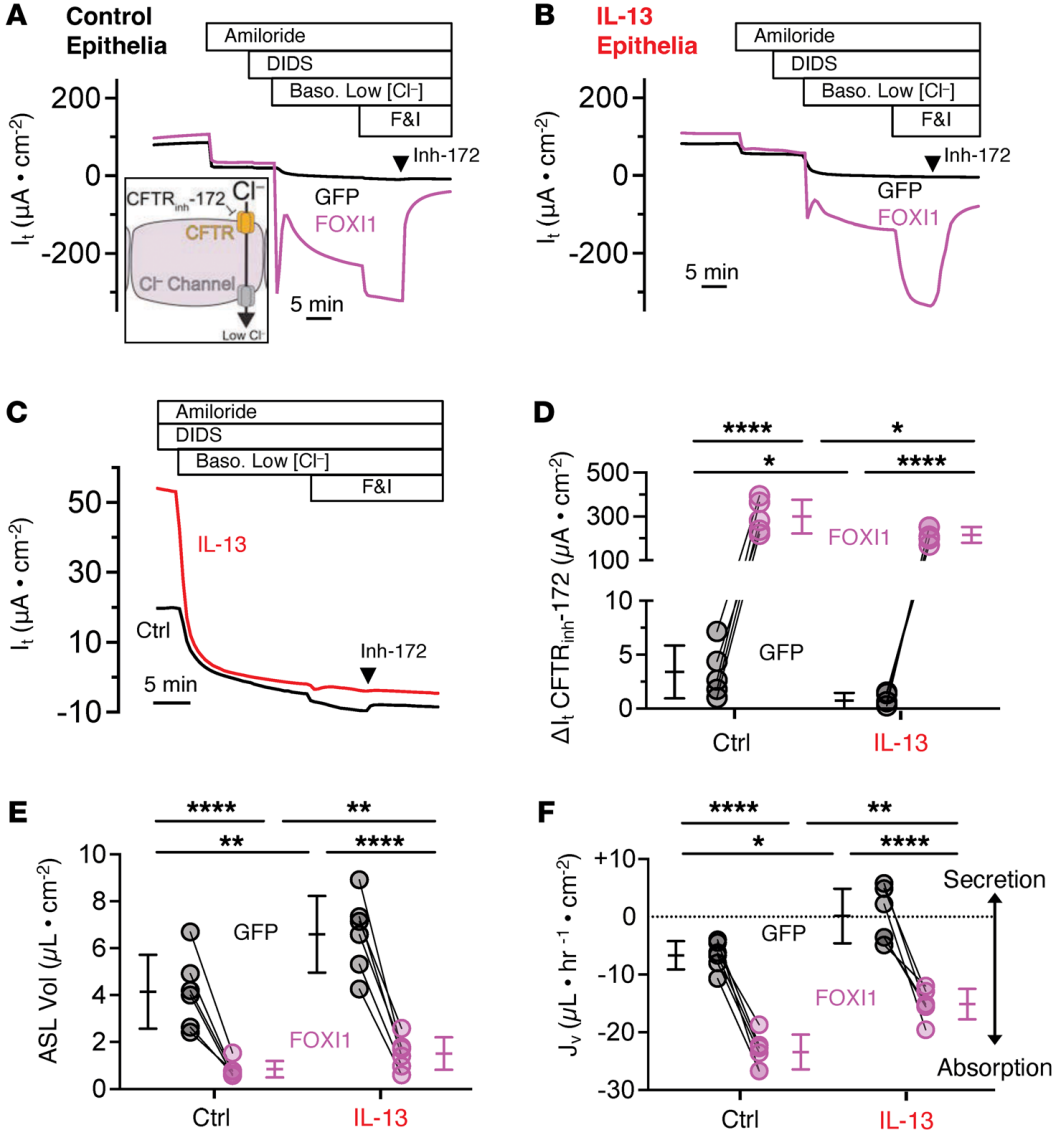
Increasing ionocyte abundance limits IL-13–induced liquid secretion. Human airway epithelia were transduced with a lentivirus expressing GFP or FOXI1 and GFP, to generate and label ionocytes. Epithelia were then treated with vehicle control or IL-13 for 24 days. (**A**–**C**) Representative transepithelial current (*I*_t_) tracings. To drive passive Cl^–^ current into cells through apical CFTR channels and out through basolateral barttin/Cl^–^ channels, transepithelial voltage was held at 0 mV and the basolateral [Cl^–^] was reduced (depicted in inset). (**A**) No IL-13 treatment (control). (**B**) IL-13 treatment. (**C**) Focus on CFTR_inh_-172–sensitive *I*_t_ generated by GFP controls from **A** and **B**. (**D**) CFTR_inh_-172–sensitive *I*_t_ summary data. (**E**) ASL volume of human airway epithelial cultures. (**F**) Liquid flux (*J*_v_) in epithelial cultures. Data points connected by a line represent paired experiments from a single human donor. Data are shown as mean ± SD. *P* values were obtained by a 2-way ANOVA with Šidák’s multiple-comparison test. Baso, basolateral; Ctrl, control; DIDS, 4,4′-dilsothiocyano-2,2′-stilbenedifulonic acid; F&I, forskolin and 3-isobutyl-1-methylxanthine; GFP, green fluorescent protein; Inh-172, CFTR_inh_-172; *I*_t_, transepithelial current. *P < 0.05; **P < 0.01; ****P < 0.0001.

We hypothesized that reintroducing ionocytes to IL-13–treated epithelia would increase ASL absorption. Forkhead box I1 (FOXI1) overexpression increases ionocyte number and liquid absorption in airway epithelial cultures (29, 30, 33, 39). Using viral vectors, we expressed GFP or FOXI1-GFP, then treated airway epithelia with IL-13 (Figure 5A). We found that FOXI1 overexpression increased the number of barttin-positive, NGFR-positive ionocytes about 100-fold in both control and IL-13–treated epithelia (Figure 5, B–D). Using CF epithelia, we previously reported that ionocytes provide a CFTR-dependent pathway for passive Cl^–^ absorption across the apical membrane (33). We performed a similar series of experiments after increasing the number of ionocytes and treating the epithelia with vehicle or IL-13. Under transepithelial voltage-clamp conditions (V_clamp_ = 0), epithelia were treated with apical amiloride and apical 4,4′-dilsothiocyano-2,2′-stilbenedifulonic acid (DIDS) to isolate CFTR-dependent ion flow. Then we bathed the basolateral surface of epithelia in low-[Cl^–^] solutions to drive Cl^–^ movement from the apical to the basolateral chamber. Finally, we revealed CFTR-mediated apical-to-basolateral Cl^–^ flow by treating epithelia with forskolin/IBMX and the CFTR inhibitor CFTR_inh_-172 (Figure 6, A–C) (33). Compared with control epithelia, CFTR_inh_-172 blocked less transepithelial current (*I*_t_) in IL-13–treated epithelia (Figure 6, A–D), consistent with our earlier findings that IL-13 treatment reduced ionocyte number and ionocyte CFTR expression. In epithelia overexpressing FOXI1, we saw a significant increase in the CFTR-dependent *I*_t_ in the absence or presence of IL-13 (Figure 6D). Finally, increasing the number of ionocytes, in both control and IL-13–treated epithelia, significantly decreased ASL volume and increased the liquid absorption rate (Figure 6, E and F). These results indicate that a decrease in ionocyte-mediated ASL absorption contributes to the IL-13–mediated increase in ASL volume.

### Ionocyte CFTR expression is reduced in IL-13–associated human airway diseases.

**Figure 7:**
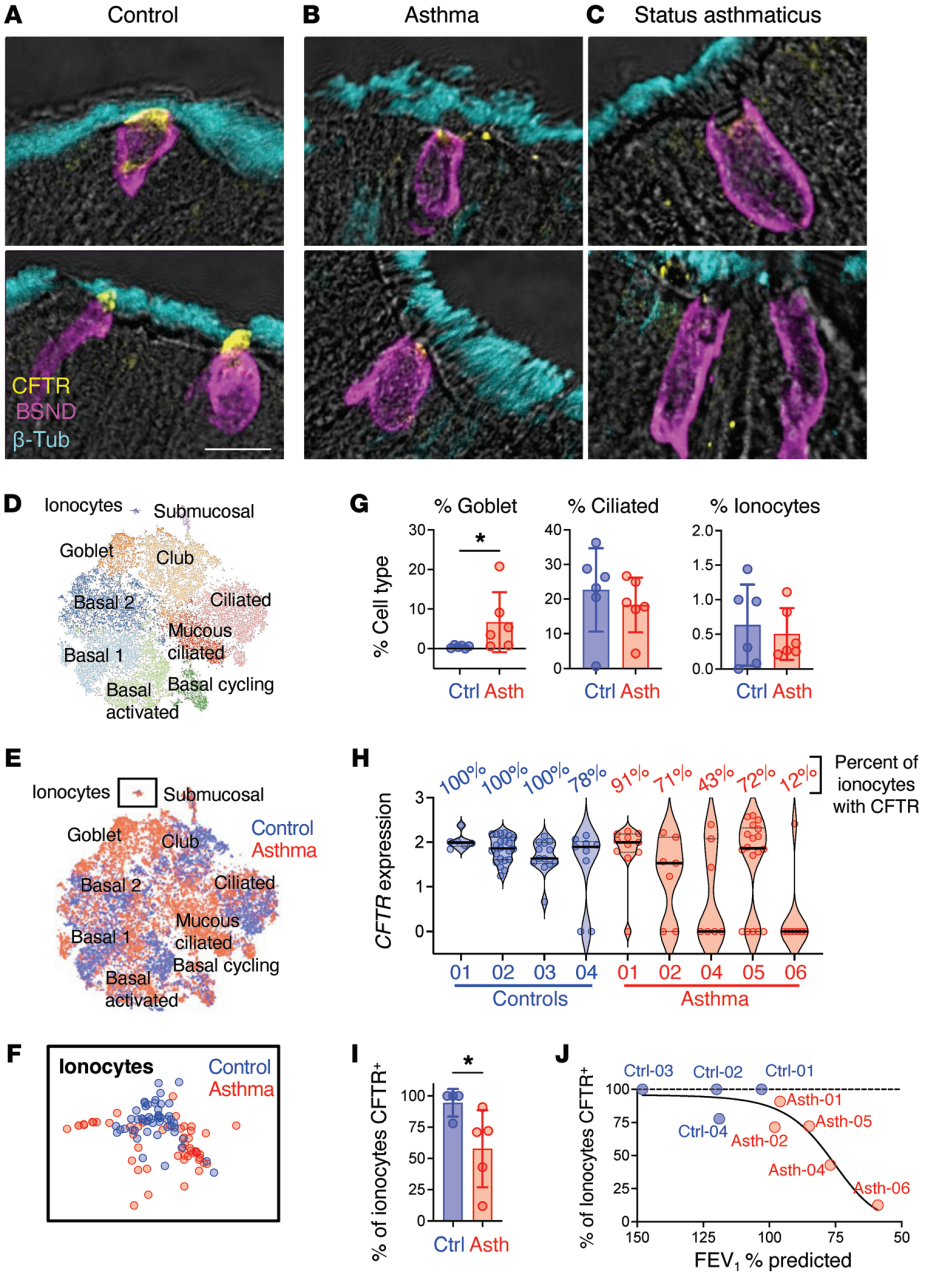
CFTR loss in ionocytes is associated with worsening airflow obstruction in asthmatic subjects. (**A**–**C**) Lung histology sections from deceased human donors stained with CFTR (yellow), barttin (magenta, ionocytes), and β-tubulin (cyan, ciliated cells). Sections were obtained from a control without known lung disease (**A**) and subjects with a history of asthma (**B**) and status asthmaticus (**C**). Scale bar: 15 μm. (**D**–**J**) scRNA-Seq and pulmonary function data are from Vieira Braga et al. (42). Data are from 6 asthmatic subjects and 6 age-matched controls. scRNA-Seq data were obtained from tracheal biopsies. The authors’ cluster definitions were preserved for this analysis. (**D** and **E**) t-SNE plots with cell type clusters for control and asthmatic subjects. (**F**) Ionocyte cluster. (**G**) Relative contributions of cell types per subject (individual symbols) by cell cluster identity. (**H**) Normalized ionocyte CFTR expression for each subject. Within a subject, individual ionocytes are each represented by a symbol. The percentage of ionocytes with CFTR was evaluated for subjects with more than 3 ionocytes. (**I**) Percentage of ionocytes with CFTR. Each symbol represents a different subject. (**J**) The percentage of CFTR-positive ionocytes was plotted versus FEV_1_% predicted for control and asthmatic subjects. The black line represents a nonlinear best-fit model. Data are shown as mean ± SD. *P* values were obtained by 2-tailed Mann-Whitney ranking test comparing asthmatic donors with control donors. Asth, asthma; Ctrl, control; FEV_1_, forced expiratory volume in 1 second. *P < 0.05.

We found that IL-13 treatment caused significant changes in ionocyte number and CFTR expression. Thus, we hypothesized that ionocytes in human airway diseases with elevated IL-13 would also exhibit altered CFTR detection. Asthma and COPD are both heterogeneous diseases, with their phenotypes associated with IL-13 (11, 12, 40, 41). To test our hypothesis, we performed immunofluorescence microscopy on formalin-fixed lung sections from deceased donors. We obtained lung sections from 1 control without lung disease, 2 COPD donors, a donor with asthma, and a donor who died from an asthma exacerbation (status asthmaticus). We stained serial sections for MUC5AC and tubulin, to look for evidence of goblet cell metaplasia, or for barttin and CFTR. In the control donor, we consistently observed strong CFTR labeling at the apical surface of barttin-positive ionocytes (Figure 7A). In COPD and asthma samples, we found an increased number of MUC5AC-positive cells (goblet cell metaplasia), consistent with elevated exposure to IL-13 (Supplemental Figure 4). However, we did not detect apical CFTR on most ionocytes but detected CFTR staining on other cells (Figure 7, B and C, and Supplemental Figure 5). These findings are consistent with our results from IL-13–treated human airway epithelia.

To further investigate ionocyte *CFTR* expression in asthma, we analyzed previously published single-cell RNA sequencing (scRNA-Seq) data obtained from lower-airway biopsies of asthma patients or controls (Figure 7, D–F) (42). We preserved the cluster classifications designated by the original authors. The clustering revealed more goblet cells in people with asthma compared with controls; however, ionocytes occurred at a similar frequency (Figure 7, E–G). We next determined the mean expression of *CFTR* per ionocyte for each participant. We restricted our analysis to samples with more than 3 ionocytes, which excluded 2 controls and 1 person with asthma. Nearly all control ionocytes contained *CFTR* (Figure 7H). In contrast, ionocytes from people with asthma appeared as 2 populations: one population expressed *CFTR* mRNA at levels comparable to controls, while the other population of ionocytes lacked *CFTR* mRNA (Figure 7H). Overall, the percentage of ionocytes with CFTR was reduced in asthmatic samples (Figure 7I). Interestingly, when we compared the number of ionocytes with detectable *CFTR* in a participant with their spirometry, we found that the CFTR-positive ionocyte number fell with worsening airflow obstruction (Figure 7J). These data suggest that in asthma and other IL-13–driven diseases the absorption property of ionocytes involving CFTR and barttin/Cl^–^ channels could be affected, while the total number of ionocytes might remain unchanged.

## Discussion

We found that IL-13 has divergent effects on CFTR expression/function in different types of airway epithelial cells, secretory cells versus ionocytes. We and others have shown that IL-13– and/or IL-4–mediated Th2 airway inflammation increases goblet cell number, *CFTR* expression, and Cl^–^ secretion and is accompanied by increased ASL volume (19–21, 25, 27, 43–49). The ionocyte’s role in this response has been unknown. Unexpectedly, we discovered that IL-13 decreases the number of CFTR-rich barttin-positive ionocytes and eliminates apical CFTR detection in the remaining ionocytes. Thus, IL-13 increases ASL volume by both increasing Cl^–^ secretion, through secretory cells, and inhibiting Cl^–^ absorption, through ionocytes.

The discovery of ionocytes has prompted multiple groups to consider how CFTR is differentially regulated in different cells (33, 39, 50, 51). These studies underlie the idea that CFTR has opposing functions depending on the cell type that expresses it. This current study furthers the idea that CFTR performs Cl^–^ secretion in secretory cells and contributes to fluid absorption in ionocytes. By challenging epithelia with IL-13, we demonstrated that the same agonist has opposing effects on CFTR regulation in a cell type–specific manner. IL-13 increases the contribution from secretory cells to epithelial CFTR function, while decreasing the contribution from ionocytes to epithelial CFTR function. These changes were associated with an increase in ASL volume. How specific cell types differentially regulate CFTR expression remains to be determined, but could have therapeutic implications.

The mechanism(s) underlying reduced ionocyte CFTR expression following IL-13 treatment are unknown. Sonic hedgehog (SHH) signaling was recently discovered to play a key role in ionocyte specification (52). Inhibition of SHH signaling reduced both the total number of barttin-positive ionocytes and the fraction of barttin-positive cells that were CFTR-positive; these results are similar to what we found with IL-13 treatment. However, several studies found that IL-4/IL-13 enhances SHH signaling, which would not fit with our findings. Thus, whether changes in SHH signaling account for our results is unknown and could depend on differences in cell culture models, in vitro versus in vivo conditions, and/or acute versus chronic treatments. While we focused our study on the effect of IL-13 on CFTR in ionocytes, it will be important in the future to investigate the effect of IL-13 on other ionocyte-specific proteins, including ATP6v0d2, and the transcription factors FOXI1 and ASCL3 (29, 30, 52).

IL-13 is increased in patients with airway disease, including asthma and COPD (11, 12, 40, 41). To determine the relevance of our in vitro findings for human disease, we used 2 approaches. First, we obtained lung histological samples from patients with COPD, asthma, and status asthmaticus. We hypothesized that ionocytes in Th2-diseased lungs would have reduced CFTR levels. In healthy control lungs, we consistently found that barttin-positive ionocytes had strong CFTR immunofluorescence at the apical surface. In contrast, in all 4 donors with airway disease, barttin-positive ionocytes lacked detectable CFTR immunofluorescence. This was associated with an increase in goblet cell abundance in all donor samples. Second, to further investigate how IL-13 might alter ionocyte morphology in the context of disease, we explored published scRNA-Seq databases. We found that asthmatic patients had higher goblet cell proportions, and that ionocytes from asthmatic donors were more likely to lack *CFTR* than ionocytes from healthy controls. How losing CFTR expression on ionocytes alters airway physiology or contributes to disease is unknown. However, we found an association between the proportion of ionocytes with CFTR in asthmatic patients and airflow obstruction. Whether the loss of ionocyte CFTR is contributing to worsening pulmonary function or simply reflects a more advanced disease state is unknown and requires further study.

The degree to which ionocytes contribute to ASL volume regulation could vary by region, and upper versus lower airway disease may differentially impact ionocyte function. Scudieri et al. found a “proximal-to-distal” gradient of ionocytes with more ionocytes present in nasal versus bronchial samples (53). In the nasal mucosa of children with chronic rhinosinusitis, Han et al. found that the number of CFTR-positive ionocytes was reduced (54). These data suggest that the changes we observed could occur in both upper and lower airways.

The therapeutic implications of our findings are unknown. However, this work raises several questions. First, in the setting of increased mucus secretion, is it beneficial or harmful to switch from an absorptive to secretory epithelium? Earlier work from Galietta and colleagues demonstrated that bicarbonate secretion is required for effective mucin release in IL-4–treated airway epithelial cells (26), suggesting that a secretory phenotype is likely beneficial in the setting of goblet cell hyperplasia. Second, do biologics that target the IL-4/IL-13 pathway, in asthma or COPD, affect the number of CFTR-rich barttin-positive ionocytes (55, 56)? And if so, are there therapeutic effects? Finally, does this work have implications for CF? In the absence of functional CFTR, other strategies to enhance liquid secretion could be beneficial, such as targeting other chloride channels (57, 58).

Our study has strengths and limitations. Its strengths include: (a) We optimized a multicolor flow cytometry–based approach to study epithelial populations. Other groups have published techniques for using flow cytometry on airway epithelial cells (59, 60). We were able to capture the diverse range of cells in airway epithelia. Our technique has the distinct advantages that it uses markers commonly used in airway biology; results in discrete, mutually exclusive populations; and is scalable for high-throughput experimentation. (b) Flow cytometry is well suited to the study of ionocytes. The low abundance of ionocytes presents a challenge for studying the effect of interventions on ionocyte populations. Current scRNA-Seq technology limits the number of cells that can reasonably be profiled. Available data sets usually contain only a handful of ionocytes per sample, amplifying noise from the stochastic selection of cells. On the other hand, microscopy-based analyses are slow and labor-intensive. Flow cytometry allowed us to quickly measure the abundance of basal cells, ciliated cells, goblet cells, secretory cells, and ionocytes. Importantly, we were able to observe approximately 10^4^–10^5^ cells per sample, allowing us to observe approximately 10^2^–10^3^ ionocytes per replicate. This greatly increased the power of our study and allowed us to detect a 2-fold reduction in ionocytes after IL-13. (c) The effect of IL-13 on ASL volume was measured, leaving the ASL intact. (d) The effect of IL-13 on transepithelial liquid absorption, which includes both electrogenic and electroneutral processes, was measured. (e) We complemented our in vitro findings with scRNA-Seq and spirometric data from humans with asthma. (f) We confirmed our flow cytometry findings by immunofluorescence confocal microscopy, detecting the reduction in barttin-positive ionocytes in IL-13 cultures, and highlighting how both flow cytometry and confocal microscopy can be complementary techniques for studying epithelial populations.

This study also has important limitations: (a) We limited our experiments to human airway epithelia. Effects of IL-13 on ionocyte biology could diverge among species. (b) While our flow cytometry analyses indicated a decrease in barttin-positive ionocytes after IL-13 treatment, it is interesting to note that scRNA-Seq studies observe similar numbers of ionocytes in Th2-driven diseases (42, 61). These differences may arise from using barttin as a marker for ionocytes instead of the ionocyte transcriptome. In addition to regulating CFTR, barttin may also be specifically reduced. If CFTR-negative, barttin-negative ionocytes are closer transcriptionally to “healthy” ionocytes than other epithelial cells, the t-distributed stochastic neighbor embedding (t-SNE) analysis will still assign them to the ionocyte cluster. (c) Vieira Braga et al. (42) only published normalized expression values, so we were unable to determine the sequencing depth for each cell. If ionocytes from asthmatic donors had lower sequencing depths, it would increase the likelihood that *CFTR*-negative ionocytes are false negatives.

Our study suggests that the hypersecretory phenotype seen in Th2-driven airway disease is mediated, in part, by opposing effects on CFTR. On one hand, IL-13 increases CFTR on secretory cells to increase Cl^–^ secretion. At the same time, IL-13 reduces CFTR on barttin-positive ionocytes and reduces their abundance, decreasing Cl^–^ absorption. Together, both mechanisms contribute to liquid secretion. Our findings suggest that precise targeting of CFTR in specific cells could have important therapeutic implications.

## Methods

### Sex as a biological variable.

Human airway epithelial cultures were randomized and studied based on availability. Epithelia from both sexes were used for these data sets.

### Airway epithelia culture.

Mature cultures were established and maintained as previously described (62). Mature epithelia were dissociated with Accutase (STEMCELL Technologies) for 30–60 minutes and resuspended in 400 μL of PneumaCult Ex+ buffer (STEMCELL Technologies) per epithelial insert. Two hundred microliters (about 5 × 10^4^ cells, a 1:2 seeding ratio) was added to 0.33 cm^2^ Corning Transwells that had been previously coated with type IV collagen with 400 μL PneumaCult Ex+ basolaterally. Cultures were incubated at 37°C and 5% CO_2_ for 24–36 hours, at which point apical medium was removed by gentle pipetting of the apical surface to remove non-adherent cells from the filter and replaced with fresh PneumaCult Ex+; basolateral medium was replaced with Ultroser G (USG) (Sartorius). Twenty-four to thirty-six hours later, apical medium was removed and cells were cultured at the air-liquid interface (ALI). Basolateral USG was changed every 2–3 days. IL-13 was reconstituted in PBS lacking divalent cations (PBS^–/–^) and used at 20 ng/mL.

### Electrophysiology.

After 3 weeks of culturing at the air-liquid interface, we studied epithelia using voltage-clamp technique and Ussing chambers. Each epithelium was bathed in a 37°C, pH 7.40 Krebs solution containing (in mM): 135 NaCl, 2.4 K_2_HPO_4_, 0.6 KH_2_PO_4_, 1.2 CaCl_2_, 1.2 MgCl_2_, 5 HEPES, and 5 glucose. Transepithelial voltage-sensing electrodes and current-passing electrodes were constructed with 3 M KCl-agar bridges and offset using a mounted culture insert without cells before mounting an epithelium. The empty culture insert was then replaced with an epithelial culture. Once the transepithelial voltage across the epithelial culture stabilized, the transepithelial voltage was held at 0 mV with an operational amplifier (VCC-MC8, Physiologic Instruments), and short-circuit current (*I*_sc_) was recorded using Acquire and Analyze software (Physiologic Instruments). Transepithelial conductance was periodically monitored by transiently holding the transepithelial voltage at –5 mV, then +5 mV, and recording the resultant changes in current. After the electrophysiology experiment, the voltage clamp was released, and each chamber was washed with 20 mL of fresh 37°C Krebs solution, then dismounted for analysis by flow cytometry.

### Flow cytometry.

Epithelial cultures were washed using PBS^–/–^, stained with Fixable Near IR or Fixable Yellow Viability dyes (Thermo Fisher Scientific) before dissociation, and then incubated with 400 μL of Accutase (200 μL apical, 200 μL basolateral) for 30–60 minutes at 37°C. Accutase was diluted by addition of 400 μL PBS^–/–^ basolaterally. The single-cell suspension was generated by vigorous pipetting of the apical liquid through a P200 tip, then transferred to a 1.5 mL microcentrifuge tube. The basolateral liquid was subsequently used to wash the apical membrane in approximately 150 μL volumes, totaling 3–4 washes. The microcentrifuge tube was then spun for 1 minute at 1,000 RCF to pellet the single-cell suspension. Single-cell suspensions were fixed using the FOXP3 Fixation and Permeabilization Kit (eBioscience/Thermo Fisher Scientific) following the manufacturer’s protocols. Cells were stored in Flow Buffer (Permeabilization Buffer) supplemented with 10% Superblock (Thermo Fisher Scientific) and 1% normal goat serum (Jackson ImmunoResearch Laboratories). Cells from individual epithelia were stored in a volume of 500 μL. Single-cell suspension (100–250 μL) stored in Flow Buffer was transferred into a 96-well V-bottom plate. Cells were spun down and resuspended in Flow Buffer supplemented with the desired conjugated antibodies and mixed 2–3 times using a P200 multichannel pipette. The plate was incubated at 37°C for 20–30 minutes, and then washed 3 times and resuspended with Flow Buffer. Samples were analyzed on a 2019 4-laser Attune NxT Flow Cytometer (Thermo Fisher Scientific) using an autosampler in the 96-well V-bottom plate used for sample preparation. The Attune is an injection-based system that allows for the calculation of cell counts in a given volume. Single-color controls were generated using AbC Total Compensation beads (Thermo Fisher Scientific). Flow cytometry standard (FSC) files were exported, compensated, and analyzed using FlowJo v10 (FlowJo LLC). Antibodies are listed in Supplemental Table 1.

### Quantitative reverse transcriptase PCR.

RNA was isolated from human airway epithelial cultures using the RNeasy kit (QIAGEN). Two epithelia for each condition were suspended in 30 μL Buffer RLT, and then RNA was isolated per the manufacturer’s instructions. A total of 500 ng RNA was used to generate DNA libraries using SuperScript VILO MasterMix (Invitrogen) per the manufacturer’s instructions. Relative expression was analyzed using a QuantStudio 6 Real Time system (Thermo Fisher Scientific). Primer pairs used include *BSND* (forward: 5′-CACCCAGCCATTTTTGGC; reverse: 5′-AGGAGCGATCCACACGAA), *CFTR* (forward: 5′-CACCCAGCCATTTTTGGC; reverse: 5′-AGGAGCGATCCACACGAA), and reference transcript *RPL13A* (forward: 5′-GGCCCCTACCACTTCCG; reverse: 5′-ACTGCCTGGTACTTCCA).

### Fluorescence in situ hybridization.

Primary human airway cultures were fixed with cold 4% paraformaldehyde (PFA) solution (Thermo Fisher Scientific) for 24 hours. Airway cultures were washed with PBS and dehydrated with 30% sucrose overnight. Airway culture filters were embedded with Tissue-Plus OCT Compound (Thermo Fisher Scientific). Ten-micron-thick sections were cut and subjected to a single-molecule fluorescence in situ hybridization method named proximity ligation in situ hybridization (63) with slight modifications (64). In brief, tissue sections were fixed in PFA, incubated in 1× citric buffer with 0.05% lithium dodecyl sulfate (Sigma-Aldrich) and 0.1% Triton X-100 at 65°C for 30 minutes, and digested with 0.05 mg/mL pepsin (Sigma-Aldrich) in 0.1 M HCl. Tissue sections were then sequentially incubated with synthesized oligonucleotide probes including gene-specific hybridization probes and circularization probes (63). Next, tissue sections were treated with circularization ligation solution with T4 DNA and rolling circle amplification solution with Lucigen’s NxGen phi29 DNA Polymerase (VWR), and fluorescently labeled with label probes (63). Finally, tissue sections were mounted in antifade mounting medium with DAPI and imaged with Olympus confocal microscopy (FV-3000). The short cDNA (mRNA) sequences targeted by the paired hybridization probes were as follows: *ASCL3*: 5′-GGCCCCGGTGTCATCCCCTTACTCTGAGGAGCTGCCACGG-3′, 5′-CTCTCTTATCCTGGGAAATTACAGTGAACCCTGCCCCTTC-3′, 5′-CAGAGGGTGCGAGTACTCCTACGGGCCAGCCTTCACCCGG-3′, 5′-TTGAGTTGCTGTTTCCAAATAGAAATGAATAATATCACAA-3′; *CLCNKB*: 5′-TTGGCTTCATCAGGAACAATAGGTTCAGCTCCAAACTGCT-3′, 5′-TCTTTGTCTATGGAGCTGCTATCGGGCGCCTCTTTGGGGA-3′, 5′-TCCCATCATGCCAGGGGGGTATGCTCTGGCAGGGGCTGCA-3′, 5′-TCCCAGATCCTGGTGGGCATAGTGCGAAGGGCCCAGCTGG-3′; *CFTR*: 5′-CGCCTGGAATTGTCAGACATATACCAAATCCCTTCTGTTG-3′, 5′-CTCTTACTGGGAAGAATCATAGCTTCCTATGACCCGGATA-3′, 5′-ATCGCGATTTATCTAGGCATAGGCTTATGCCTTCTCTTTA-3′, 5′-AAGCTGTCAAGCCGTGTTCTAGATAAAATAAGTATTGGAC-3′.

### Immunofluorescence microscopy.

Individual epithelia were washed with filtered PBS, fixed using 4% PFA (Electron Microscopy Sciences) for 15 minutes, and permeabilized using 0.3% Triton X (Sigma-Aldrich) for 20 minutes. Cultured epithelia were blocked using Superblock (Thermo Fisher Scientific) supplemented with 5% normal goat serum (Jackson ImmunoResearch Laboratories) for at least 2 hours. Primary antibodies were resuspended in blocking buffer and added to epithelia for 2 hours before being washed. Conjugated Alexa Fluor antibodies (Thermo Fisher Scientific) were resuspended in blocking buffer at 1:1,000 and added to epithelia for 45 minutes before being washed off. Finally, epithelia were mounted onto slides using Vectashield Hardset mounting medium with DAPI (Vector Laboratories) and imaged using a confocal microscope (Olympus Fluoview Fv3000). Antibodies are listed in Supplemental Table 1. For human tissue sample studies, we performed immunofluorescence microscopy on formalin-fixed lung sections from deceased donors obtained through the University of Iowa Comparative Pathology Laboratory.

### Liquid absorption assay.

Liquid absorption across epithelia treated with forskolin (10 μM) and 3-isobutyl-1-methylxanthine (IBMX) (100 μM) was measured as previously described (33, 36, 37). A total of 60 μL saline buffer (in mM: 137.8 NaCl, 4 KCl, 29 NaHCO_3_, 1.2 CaCl_2_, 0.6 MgCl_2_, and 1 NaH_2_PO_4_) was added to the apical surface and incubated at 37°C and 5% CO_2_ for 4 hours, and then the remaining apical volume was collected and measured.

### ASL volume.

ASL volumes were estimated as previously described (33, 65). Briefly, cultured epithelia were treated overnight with 10 μM forskolin and 100 μM IBMX and imaged the following day. Bright-field images were acquired. For analysis, the meniscus intensity versus distance was integrated and compared with a calibration curve.

### Lentiviral transduction.

Transduction of GFP or FOXI1-GFP lentiviral vectors was performed as described in ref. 33. Briefly, the cell suspension was transduced with an HIV-based VSV-G pseudotyped lentivirus (MOI = 4) and hexadimethrine bromide (Polybrene) at a final concentration of 2 μg/mL. The cell-virus mixture was then seeded onto collagen-coated, semipermeable membranes (0.33 cm^2^, 3413 polycarbonate, Corning Costar Transwell 16 Permeable Supports) and differentiated at the air-liquid interface.

### Reanalysis of single-cell RNA sequencing data.

Data were freely obtained from the University of California, Santa Clara, Cell Browser digital atlas and analyzed on R-Studio using the Seurat package (66). Cells within the ionocyte cluster were exported and statistical analysis performed with GraphPad Prism 10.

### Statistics.

Statistical analyses were performed using GraphPad Prism 10. For some data, a Bartlett’s test revealed differences in variance among groups. In these cases, we performed statistical analyses on log transformations provided in the Supporting Data Values file. Individual statistical tests are noted in each figure. *P* less than 0.05 was considered statistically significant.

### Study approval.

The University of Iowa Institutional Review Board approved all studies.

### Data availability.

The individual data point values for all graphs are included in the Supporting Data Values file.

## Author contributions

GSRI, LL, WY, AAP, PBM, IMT, and DAS designed research studies. GSRI, LL, WY, NDG, and IMT conducted experiments. GSRI, LL, WY, NDG, DKM, and IMT acquired data. GSRI, LL, WY, ALT, AAP, PBM, IMT, and DAS analyzed data. DKM provided reagents. GSRI, LL, WY, ALT, NDG, DKM, AAP, PBM, IMT, and DAS wrote, edited, and approved the final manuscript.

## Supplementary Material

Supplemental data

Supporting data values

## Version 1 09/10/2024

In-Press Preview

## Version 2 11/01/2024

Electronic publication
