# Supplementary material for: IL-13 induces loss of CFTR in ionocytes and reduces airway epithelial fluid absorption
Source: J Clin Invest. 2024 Sep 10;134(21):e181995. doi: 10.1172/JCI181995 (PMC11527443; doi:10.1172/JCI181995)
Supplement: Supplemental data [file jci-134-181995-s139.pdf]

SUPPLEMENTAL DATA

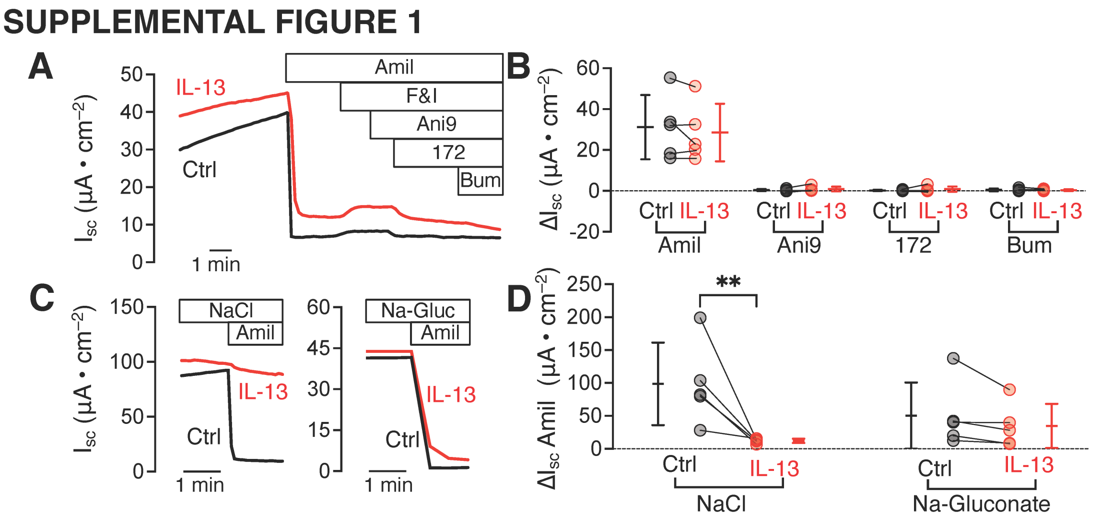

SUPPLEMENTAL FIGURE 1. Increases in Anion Permeability with IL-13 Explain Much of the Reduced Amiloride-sensitive I_{sc}. (**A**) Short-circuit current (I_{sc}) traces from CF human airway epithelial cultures exposed to vehicle control or IL-13. (**B**) Summary I_{sc} values for CF donor epithelia treated with vehicle control or IL-13 for 3-4 weeks. n = 5 donor epithelia per treatment group. (**C**) Representative I_{sc} traces from vehicle control or IL-13 treated epithelia in Cl^- containing or Cl^-- free bathing solutions and the I_{sc} response to amiloride (ΔI_{sc} amiloride). (**D**) Summary ΔI_{sc} amiloride values. Error bars represent mean ± SD. P values obtained from paired, two-sided Student's t test. Data points connected by a line represent paired experiments from a single human donor. 172, $CFTR_{inh}$-172; Amil, amiloride; Bum, bumetanide; Ctrl, control; F&I, forskolin and 3-isobutyl-1-methylxanthine.

SUPPLEMENTAL FIGURE 2

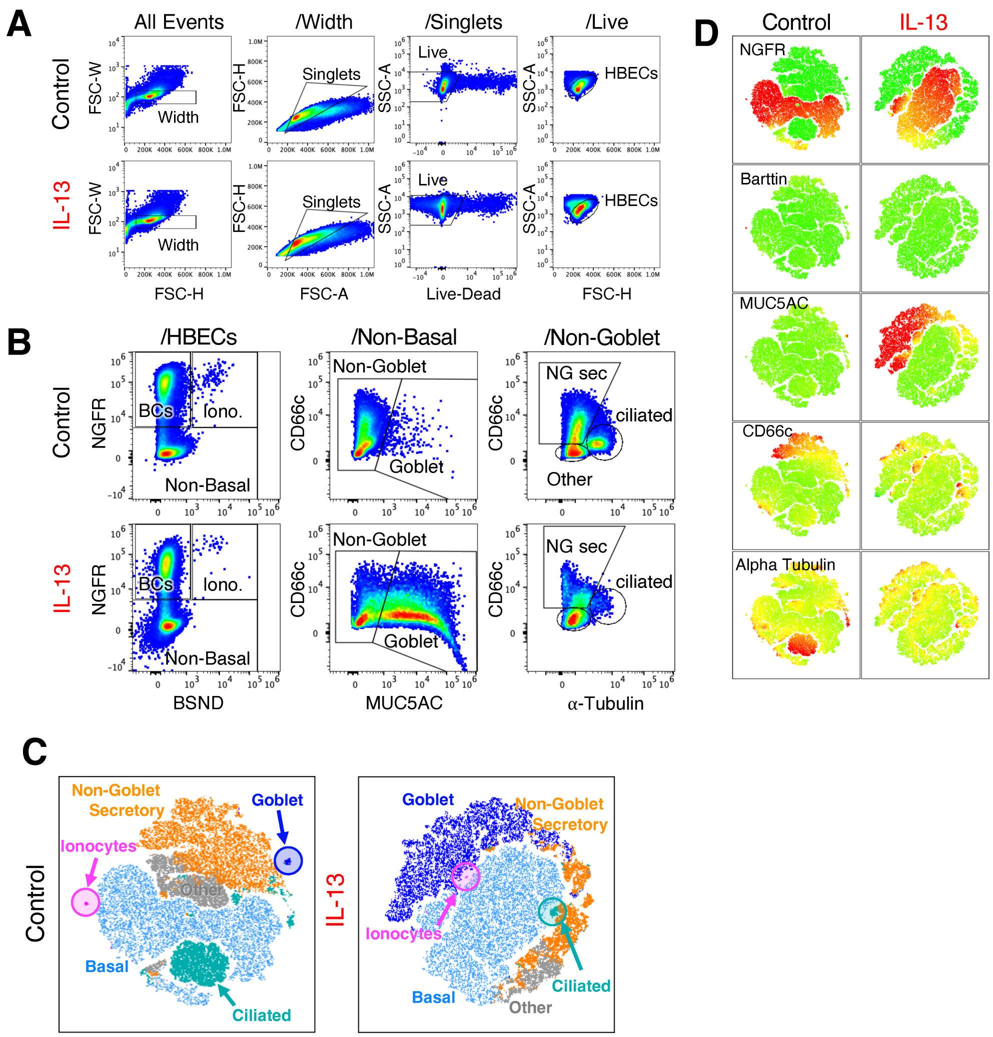

SUPPLEMENTAL FIGURE 2. Flow Cytometry Analyses of Airway Epithelia. (**A**) Gating scheme of epithelia treated with IL-13 or control. From left to right: all acquired events (/All Events) plotted by FSC-H and FSC-W, then by FSC-A and FSC-H to obtain single cells. Single cells (/Singlets) plotted by SSC-A versus Live/Dead Near-IR exclusion dye. Cells within the live gate (/Live) plotted for FSC-H and SSC-A to define human bronchial epithelial cell (HBECs) population. (**B**) Cells within the HBEC population (/HBECs) plotted by NGFR and BSND to identify basal cells (BCs), barttin-positive ionocytes (Iono.), and non-basal cells (non-basal). Cells within the non-basal cell gate (/Non-Basal) plotted for CD66c and MUC5AC to identify goblet cells and non-goblet cells. Cells within the non-goblet cell gate (/Non-Goblet) plotted for CD66c and α-tubulin to identify secretory cells, ciliated cells, or uncharacterized cells. (**C**) Unbiased tSNE analysis of the cells within the HBEC gate pseudo-colored with the gates determined in (B), used to validate manual gating scheme. (**D**) Heat map of tSNE plot in (C) representing relative fluorescent intensity for a given marker. Green represents low fluorescent intensity. Red represents high fluorescent intensity. From top to bottom: NGFR, barttin, MUC5AC, CD66c, and α-tubulin.

SUPPLEMENTAL FIGURE 3

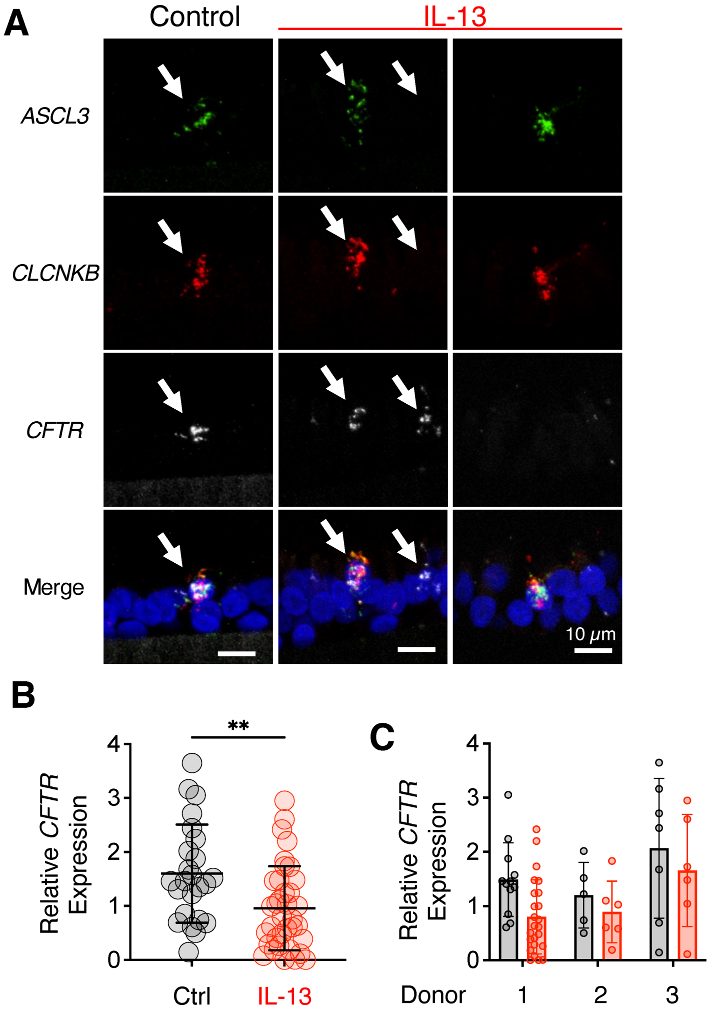

SUPPLEMENTAL FIGURE 3. IL-13 Treatment Decreases *CFTR* expression in Ionocytes. (**A**) Fluorescent in-situ mRNA hybridization (FISH) of epithelial cultures exposed to IL-13 or vehicle control. Cells were probed for the ionocyte-associated transcription factor *ASCL3,* the barttin/Cl^- channel encoding *CLCNKB,* and *CFTR*. (**B**) Relative *CFTR* expression calculated by normalizing the fluorescent intensity of *CFTR* to *ASCL3* intensity. Each symbol represents an individual ionocyte from 3 human donors. *P* value calculated from a two-tailed Student's *t* test. (**C**) Relative *CFTR* expression grouped by donor and treatment. Control (black) or IL-13 treated (red). Ctrl, control.

SUPPLEMENTAL FIGURE 4

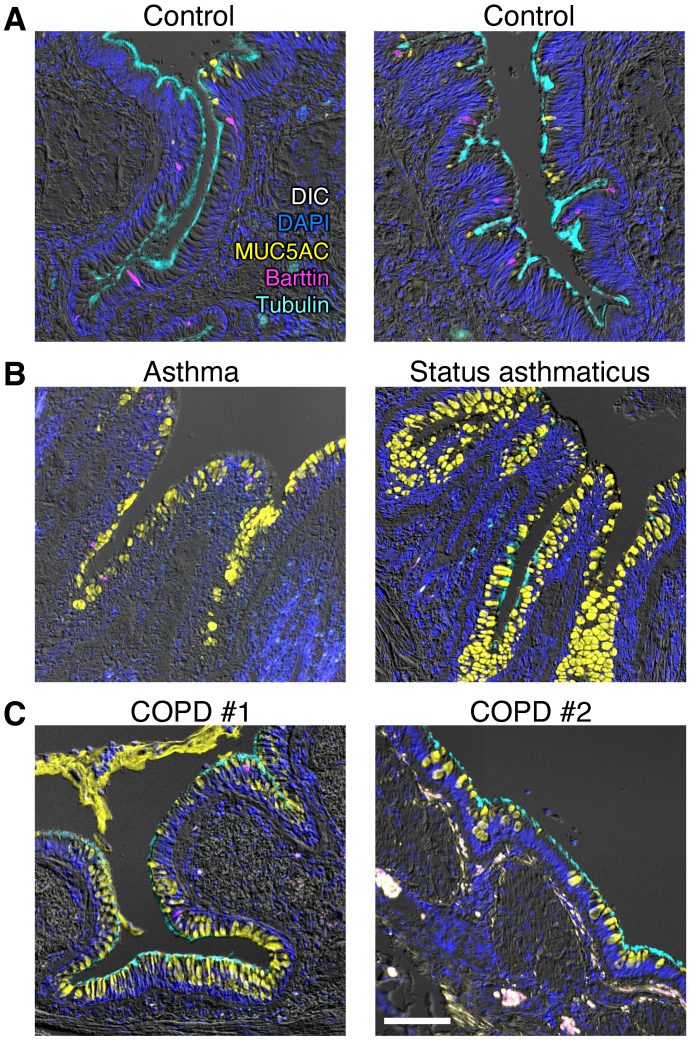

SUPPLEMENTAL FIGURE 4. MUC5AC-Positive Goblet Cell Fluorescence in Human Tissues. Immunofluorescence and imaging of serial lung sections for donor samples shown in Figure 7 and Supplemental Figure 5 for MUC5AC (yellow), tubulin (cyan), DAPI (blue), and barttin (magenta). Tissue morphology was captured by DIC microscopy. Tissues were obtained from (**A**) a control donor without airway disease, (**B**) a donor with asthma and another who succumbed to an asthma attack (status asthmaticus), and (**C**) two COPD donors. COPD, chronic obstructive pulmonary disease.

SUPPLEMENTAL FIGURE 5

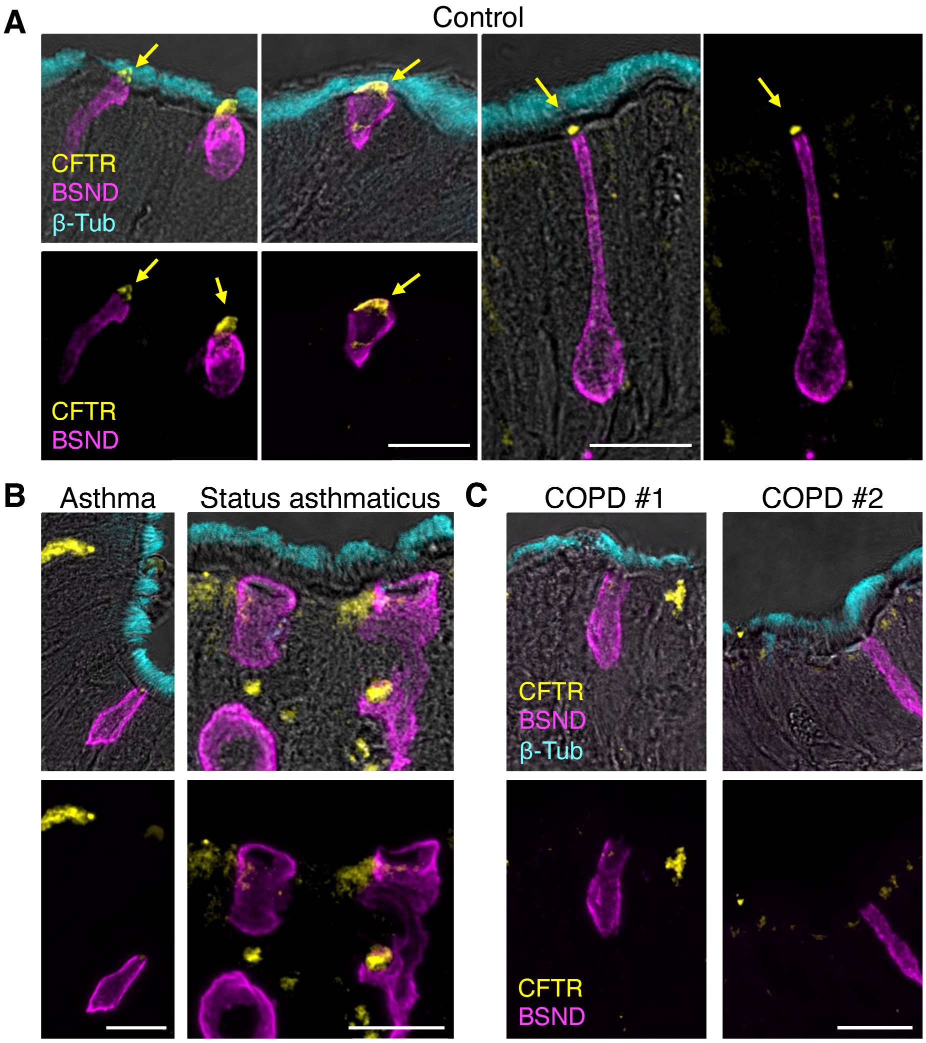

SUPPLEMENTAL FIGURE 5. Ionocytes Lacking CFTR are Found in Human Airway Diseases Associated with Increased IL-13. Lung histology sections from deceased human donors stained with CFTR (yellow), barttin (magenta, ionocytes), and β-tubulin (cyan, ciliated cells). Sections were obtained from (**A**) a control without known lung disease, (**B**) subjects with a history of asthma and status asthmaticus, and (**C**) subjects with COPD. Some images are also shown in Figure 7. Scale bar = 15 μm. COPD, chronic obstructive pulmonary disease.

SUPPLEMENTAL TABLE 1

Target	Clone	Vendor	Catalog number
alpha-tubulin	(DM1A)	Novus Biologicals	NB100-690
alpha-tubulin	(D20G3)	Cell Signaling	5335S
BSND (barttin)	(EPR14270)	Abcam	Ab196017
beta-tubulin	(TUB2.1)	Sigma	C4585-.2ML
CEACAM6 (CD66c)	(KOR-SA3544)	Invitrogen/Thermo	53-0667-42
CFTR	(769)	UNC CFTR Antibody Distribution Program	
MUC5AC	(45M1)	Novus	NBP2-32732
NGFR (CD271)	(ME20.4)	BioLegend	345110
P63	(epr5701)	Abcam	ab246728
